# Burkholderia cepacia Complex Producing a Peculiar Violet Pigment: A Case Series From a Tertiary Care Hospital in Meghalaya

**DOI:** 10.7759/cureus.64126

**Published:** 2024-07-09

**Authors:** Kunjjal Shah, Mercy Ngairangbam, Neeta Gogoi, Papia Chakraborty, W Valarie Lyngdoh

**Affiliations:** 1 Clinical Microbiology, North Eastern Indira Gandhi Regional Institute of Health and Medical Sciences, Shillong, IND

**Keywords:** emerging pathogen, vitek 2 system, nosocomial infections, critical care, bacteremia, violet pigment, burkholderia cepacia complex

## Abstract

The *Burkholderia cepacia* complex (BCC) represents a group of bacteria that are gram-negative, aerobic, and non-fermenters. They are notorious for causing infections in vulnerable individuals, such as those with compromised immune systems. Examples are patients suffering from cystic fibrosis or chronic granulomatous disease. These bacteria are prevalent in diverse habitats, like soil and water. Over the last four decades, they have gained recognition as both emerging opportunistic pathogens and nosocomial threats. Managing BCC infections poses significant challenges due to their inherent resistance to numerous antibiotics, thus raising substantial concerns within clinical settings.

Here, we present a case series of bacteremia, with BCC as the causative organism. The isolates showed a curious phenomenon of producing a violet pigment.

## Introduction

*Burkholderia cepacia* complex (BCC) is a concerning group of bacilli, characteristically aerobic, gram-negative, non-fermenters, gaining notoriety for causing infections in individuals with compromised immune systems. Examples are patients suffering from cystic fibrosis (CF) or chronic granulomatous disease. These bacteria are found widely in diverse habitats, including soil and water. They have been recognized as emerging opportunistic as well as nosocomial pathogens over the past four decades. Infections with BCC can be challenging to treat owing to their intrinsic resistance to many antibiotics, making them a significant concern in clinical settings [1].

Here, we present a report on four cases of bacteremia, with the causative organism being BCC. The isolates displayed a peculiar phenotypic characteristic of violet-pigmented colonies. Reports of this unique pigmentation in BCC are rare. However, such knowledge can contribute to the identification of non-fermenters being more comprehensive, particularly in resource-limited settings.

## Case presentation

We have recorded four cases of bacteremia caused by BCC. For each patient, we collected the relevant clinical history along with their demographic profile, including their age and sex. We also looked into whether any invasive interventions were performed for each patient. The collected data has been compiled and presented in Table 1.

**Table 1:** Background of four patients with BCC bacteremia. ICU: intensive care unit; MICU: medical intensive care unit; M: male; F: female, PCN: percutaneous nephrostomy, BCC: *Burkholderia cepacia* complex.

Sl. no.	Age/sex	Ward/ICU	Relevant clinical history	Indwelling device	Diagnosis	Surgical intervention
1	56/F	MICU	History of fever seven days after surgery, known case of diabetes mellitus (not on medication) and hypertension	Central venous catheter, Foley's catheter	Diabetic ketoacidosis with uncontrolled type 2 diabetes mellitus, dry gangrene of left foot, hypertension, mild aortic regurgitation, bilateral pleural effusion, septic shock, severe anemia	Above knee amputation of left lower limb
2	18/M	Male Surgery Ward	History of fever three days after surgery, no history of diabetes mellitus, hypertension, or tuberculosis	Foley's catheter, abdominal drain	Gastric outlet obstruction	Laparoscopic truncal vagotomy + loop gastrojejunostomy under general anesthesia
3	38/M	Urology Ward	History of fever one hour after surgery, no history of diabetes mellitus, hypertension, or tuberculosis	PCN catheter	Left renal staghorn calculus	Left percutaneous nephrolithotomy under spinal anesthesia
4	70/F	MICU	Shortness of breath, coughing on lying down, known case of diabetes mellitus and hypertension	Foley's catheter	Coronary artery disease, uncontrolled type 2 diabetes mellitus, lower respiratory tract infection, hyperosmolar hyperglycemic syndrome, septic shock, acquired cystic kidney disease	None

Paired blood samples were sent in automated blood culture bottles the same day a high temperature was recorded for each patient. Subsequently, on receipt of each sample, they were incubated in the BacT/ALERT 3D. Each sample was flagged positive, followed by their inoculation onto MacConkey agar, blood agar, and chocolate agar. Overnight aerobic incubation of the plates was done at a temperature of 37℃. The isolates that grew on culture were then identified with the VITEK® 2 system, which also determined the minimum inhibitory concentration (MIC) values for the organisms. The antimicrobial susceptibility profile was reported by interpreting the MIC values using the Clinical and Laboratory Standards Institute (CLSI) Guidelines, 2024.

**Figure 1:**
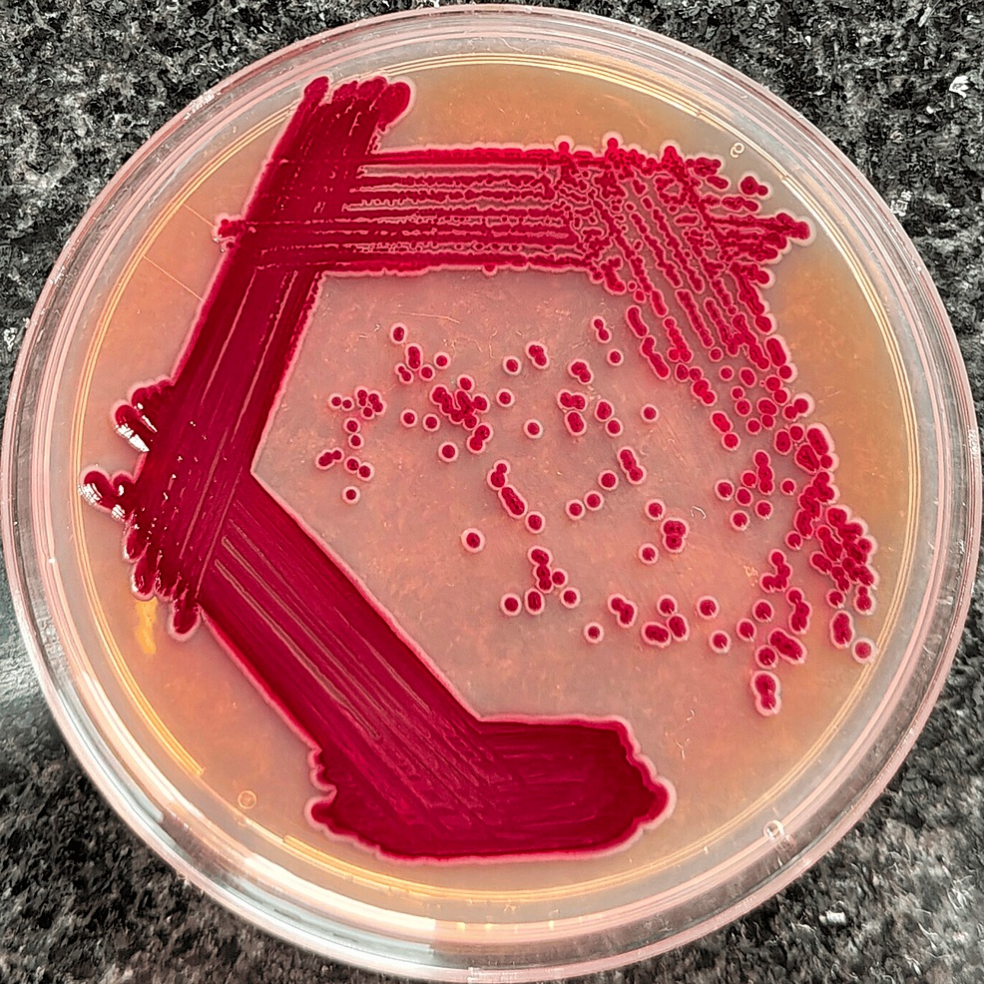
Colonies of BCC on MacConkey agar showing a violet pigmentation. BCC: *Burkholderia cepacia* complex.

**Figure 2:**
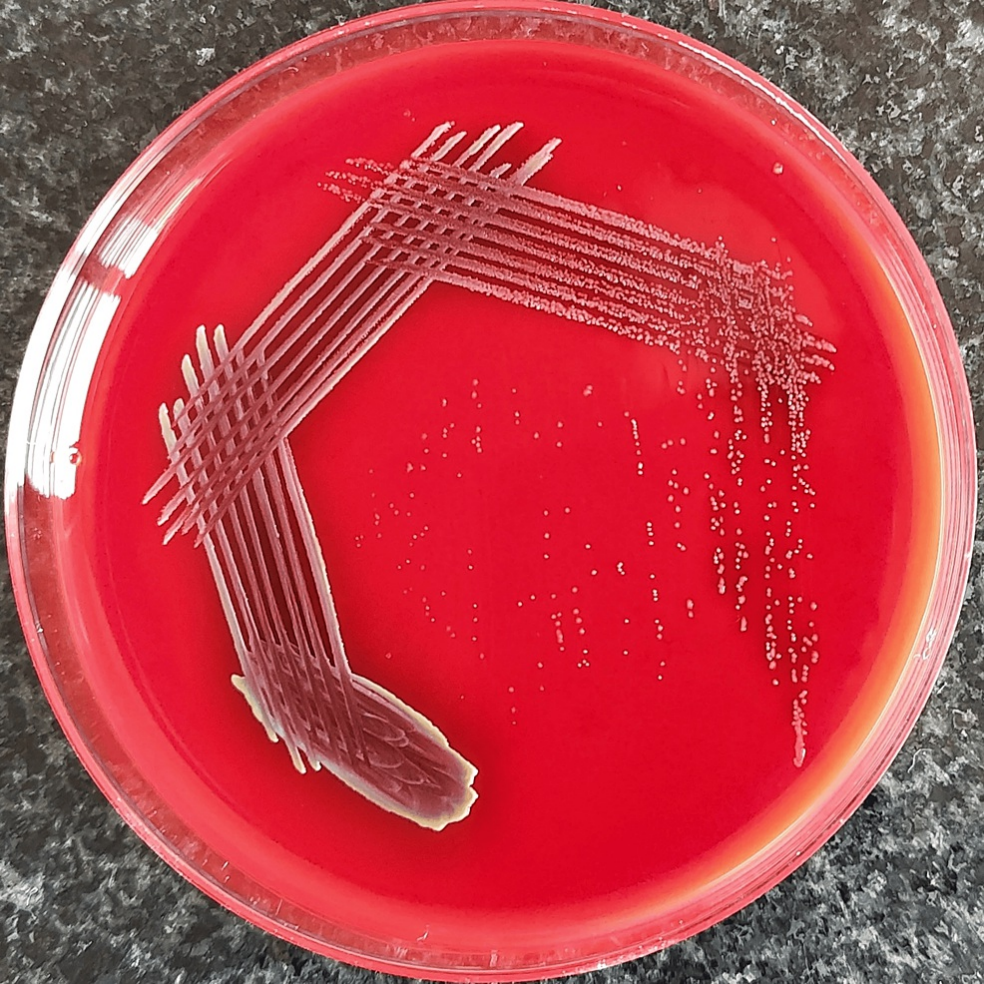
Colonies of BCC on blood agar showing black to burgundy pigmentation. BCC: *Burkholderia cepacia *complex.

**Figure 3:**
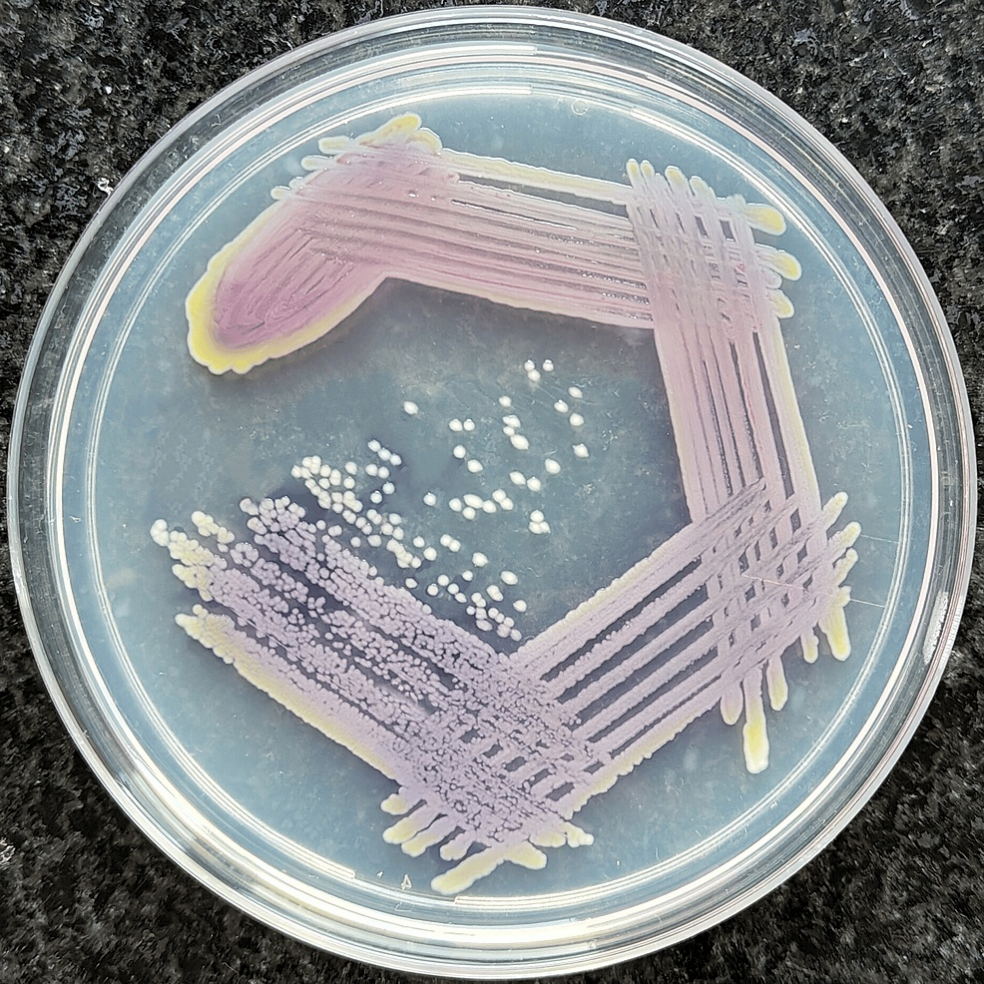
Colonies of BCC on nutrient agar showing a light violet pigmentation. BCC: *Burkholderia cepacia* complex.

The blood cultures showed growth of gram-negative bacilli, which appeared as 1-2 mm, violet-pigmented colonies on MacConkey agar (Figure 1), while 5% sheep blood agar showed growth of 0.5 mm, black to burgundy colonies with diffusible pigment of similar hue and the margins of the well showed yellow pigmentation (Figure 2). On nutrient agar, 1-1.5 mm colonies with a light violet pigment were seen, and the margins of the well showed a yellow pigmentation (Figure 3). Conventional biochemical tests revealed the organism to be catalase-positive, delayed oxidase-positive, lactose non-fermenting, and motile. Subsequent automated bacterial identification was done with the VITEK® 2 system, which established the organism as BCC. The VITEK® 2 system calculated the antibiotic susceptibility profile for the organism and interpreted it by the CLSI Guidelines, 2024. All four isolates were found to share a similar antibiotic susceptibility pattern, exhibiting sensitivity to ceftazidime, minocycline, levofloxacin, trimethoprim/sulfamethoxazole, and meropenem.

The blood cultures of the respective four cases were interspersed by weeks to months. The patients were started on levofloxacin. On completion of the antibiotic course, the BCC bacteremia was cleared, as evidenced by subsequent blood cultures.

## Discussion

The BCC comprises bacteria that share similar characteristics phenotypically but differ genetically. All members of this complex are aerobic gram-negative bacilli equipped with multiple polar flagella, granting them motility. The BCC presently comprises 22 closely related species [2]. Initially identified by Burkholder in 1940 as a cause of onion rot, the BCC demonstrates the potential for beneficial interactions with plant hosts and may serve as a valuable asset in bioremediation and as a plant-growth enhancer, partly owing to its capability of synthesizing anti-fungal agents [3-5].

In individuals with cystic fibrosis (CF), the BCC contributes to heightened mortality and morbidity, decreased lung function, and pro-inflammatory damage [6]. Typically, there are instances featuring rapid and severe pneumonic infection accompanied by fever and respiratory failure, sometimes coupled with bloodstream infection, which are referred to as "cepacia syndrome" [7].

Additionally, BCC is gaining notoriety as a nosocomial pathogen. Its capability to contaminate medical apparatus and objects, such as ultrasound gel and chlorhexidine wipes, has increasingly come to light in recent years [8-10]. Owing to its capacity to flourish in diverse habitats, BCC leads to immense mortality and morbidity among hospitalized patients. Instances of septicemia caused by BCC occur in hospitalized patients across various settings, including those who are immunocompetent or immunosuppressed, as well as in intensive care units (ICUs) and oncology units [11]. The presented cases had developed bacteremia after more than 48 hours of hospital stay, pointing to BCC being a nosocomial pathogen in these patients. In this case series, two out of four patients were in the ICU when their cultures were sent. It was also noted that three of the four cases had undergone surgical interventions, and blood cultures were dispatched during their respective episodes of fever in the postoperative period. These cultures flagged positive, suggesting the need to further investigate the source of infection, with a special focus on the operation theaters and ICUs.

The BCC demonstrates intrinsic resistance to numerous commonly employed antibiotics, including antipseudomonal penicillins (such as piperacillin, carbenicillin, and ticarcillin), aminoglycosides, polymyxins, and first- and second-generation cephalosporins [1]. These antimicrobial agents are frequently utilized in treating Pseudomonas infections, underscoring the significance of accurately distinguishing between BCC and Pseudomonas. BCC is known to display increasing drug resistance, which, however, was seen less significantly in these reported cases as the isolates showed sensitivity to ceftazidime, minocycline, levofloxacin, trimethoprim/sulfamethoxazole, and meropenem.

The isolates showed colonies having a violet pigment, which is a peculiar feature displayed by BCC. Other studies done by De et al. [12], Ranjan et al. [13], and Rastogi et al. [14] emphasize a similar phenotypical attribute of violet pigment production of BCC that they encountered. The knowledge of such peculiarities in BCC can help broaden the approach to the identification of non-fermenters, especially in resource-limited settings.

Deeper and more extensive research needs to be conducted in order to find the root cause of such phenotypical and genotypical diversity among the members of the BCC.

## Conclusions

The BCC is commonly known to be a nosocomial pathogen. We have recorded four cases where the BCC has caused hospital-acquired infection. Out of these cases, three patients shared the commonality of having surgical procedures done in the hospital. Also, out of the four patients, two had been admitted in critical care units. Rigorous surveillance and good infection prevention and control practices will aid in the prevention of nosocomial infections.

The BCC isolates from this case series displayed a rare occurrence of a violet pigment produced by these organisms. A few other studies showing similar findings warrant the need for further research into finding the root cause of such phenotypic and genotypic diversity among the BCC. The knowledge of such peculiarities in BCC is important. It can contribute to a more comprehensive approach in the identification of non-fermenters, particularly in resource-limited settings.

## References

[1] Gautam V, Singhal L, Ray P (2011). Burkholderia cepacia complex: beyond pseudomonas and acinetobacter. Indian J Med Microbiol.

[2] Devanga Ragupathi NK, Veeraraghavan B (2019). Accurate identification and epidemiological characterization of Burkholderia cepacia complex: an update. Ann Clin Microbiol Antimicrob.

[3] Vial L, Chapalain A, Groleau MC, Déziel E (2011). The various lifestyles of the Burkholderia cepacia complex species: a tribute to adaptation. Environ Microbiol.

[4] Vandamme P, Peeters C (2014). Time to revisit polyphasic taxonomy. Antonie Van Leeuwenhoek.

[5] Parke JL, Gurian-Sherman D (2001). Diversity of the Burkholderia cepacia complex and implications for risk assessment of biological control strains. Annu Rev Phytopathol.

[6] Ganesan S, Sajjan US (2011). Host evasion by Burkholderia cenocepacia. Front Cell Infect Microbiol.

[7] Jones AM, Dodd ME, Govan JR, Barcus V, Doherty CJ, Morris J, Webb AK (2004). Burkholderia cenocepacia and Burkholderia multivorans: influence on survival in cystic fibrosis. Thorax.

[8] Ko S, An HS, Bang JH, Park SW (2015). An outbreak of Burkholderia cepacia complex pseudobacteremia associated with intrinsically contaminated commercial 0.5% chlorhexidine solution. Am J Infect Control.

[9] Lee S, Han SW, Kim G (2013). An outbreak of Burkholderia cenocepacia associated with contaminated chlorhexidine solutions prepared in the hospital. Am J Infect Control.

[10] Marigliano A, D'Errico MM, Pellegrini I, Savini S, Prospero E, Barbadoro P (2010). Ultrasound echocardiographic gel contamination by Burkholderia cepacia in an Italian hospital. J Hosp Infect.

[11] Kenna DT, Lilley D, Coward A (2017). Prevalence of Burkholderia species, including members of Burkholderia cepacia complex, among UK cystic and non-cystic fibrosis patients. J Med Microbiol.

[12] De A, Shastri JS, Malak NI (2022). Unusual violet coloured pigment produced by Burkholderia cepacia complex: a report of five cases from a tertiary care hospital in Mumbai. J Med Sci Health.

[13] Ranjan R, Chowdhary P, Kamra A (2017). Community acquired Burkholderia cepacia bacteraemia presenting as MODS in an immunocompetent individual: an unusual case. J Clin Diagn Res.

[14] Rastogi N, Khurana S, Veeraraghavan B (2019). Epidemiological investigation and successful management of a Burkholderia cepacia outbreak in a neurotrauma intensive care unit. Int J Infect Dis.

